# A Pilot Study of Radiomic Based on Routine CT Reflecting Difference of Cerebral Hemispheric Perfusion

**DOI:** 10.3389/fnins.2022.851720

**Published:** 2022-03-31

**Authors:** Qingguo Ren, Panpan An, Ke Jin, Xiaona Xia, Zhaodi Huang, Jingxu Xu, Chencui Huang, Qingjun Jiang, Xiangshui Meng

**Affiliations:** ^1^Radiology, Qilu Hospital, Cheeloo College of Medicine, Shandong University, Qingdao, China; ^2^Deepwise AI Lab, Beijing Deepwise and League of PHD Technology Co., Ltd., Beijing, China

**Keywords:** cerebral ischemia, computed tomography, machine learning, middle cerebral artery, different region of interest

## Abstract

**Background:**

To explore the effectiveness of radiomics features based on routine CT to reflect the difference of cerebral hemispheric perfusion.

**Methods:**

We retrospectively recruited 52 patients with severe stenosis or occlusion in the unilateral middle cerebral artery (MCA), and brain CT perfusion showed an MCA area with deficit perfusion. Radiomics features were extracted from the stenosis side and contralateral of the MCA area based on precontrast CT. Two different region of interest drawing methods were applied. Then the patients were randomly grouped into training and testing sets by the ratio of 8:2. In the training set, ANOVA and the Elastic Net Regression with fivefold cross-validation were conducted to filter and choose the optimized features. Moreover, different machine learning models were built. In the testing set, the area under the receiver operating characteristic (AUC) curve, calibration, and clinical utility were applied to evaluate the predictive performance of the models.

**Results:**

The logistic regression (LR) for the triangle-contour method and artificial neural network (ANN) for the semiautomatic-contour method were chosen as radiomics models for their good prediction efficacy in the training phase (AUC = 0.869, 0.873) and the validation phase (AUC = 0.793, 0.799). The radiomics algorithms of the triangle-contour and semiautomatic-contour method were implemented in the whole training set (AUC = 0.870, 0.867) and were evaluated in the testing set (AUC = 0.760, 0.802). According to the optimal cutoff value, these two methods can classify the vascular stenosis side class and normal side class.

**Conclusion:**

Radiomic predictive feature based on precontrast CT image could reflect the difference of cerebral hemispheric perfusion to some extent.

## Introduction

The brain is an oxygen-consuming organ and consumes 20% of the body’s oxygen to meet its high-energy demands (Farrell et al., 2017). This physiological characteristic makes the brain the most vulnerable organ to ischemia and cerebral hypoxia, for all that the brain has a self-protective and self-repairing mechanisms against these conditions. Ischemic tolerance or preconditioning is an endogenous neuroprotective phenomenon (Koizumi et al., 2018). Such as, severe stenosis or occlusion of the internal carotid artery (ICA) may lead to cerebral ischemic damage, but the cerebral blood flow (CBF) is redistributed toward the affected hemisphere to maintain adequate perfusion, as a result, cerebral autoregulation can compensate for a fall in cerebral perfusion pressure through vasodilation (van Everdingen et al., 2000). For another aspect, occlusion or stenosis of the middle cerebral artery (MCA) is considered to be the most common vascular cause of stroke in Asian populations (Sacco et al., 1995). A recent report showed that cerebral hemodynamic disturbance can be found frequently in patients with symptomatic unilateral MCA high-grade stenosis or occlusion (Lu et al., 2007).

Computed tomography perfusion (CTP) has been a well-developed method to detect and evaluate cerebral hemodynamic disturbance and the CTP pattern downstream from cerebrovascular stenosis yields important information about the hemodynamic significance of this stenosis. This information may be more relevant than merely measuring the degree of luminal narrowing. Chronic cerebral hypoperfusion is a key mechanism of neurodegeneration including vascular cognitive impairment and dementia (Duncombe et al., 2017; Washida et al., 2019) and it could be minified by CTP. Neurodegeneration from hypoperfusion involves intracellular Ca^2+^ dyshomeostasis and excitotoxic activation of neuronal glutamate receptors (Diemer et al., 1993). Cytoskeletal disintegration occurring in dendrites and degeneration of neuronal cell bodies persist during the process of neurodegeneration (Martin et al., 1998). Any sustained reduction in regional CBF may reduce tissue function and cause regional cerebral damage even neuronal and non-neuronal cells death (Martin et al., 2000). However, patients must be exposed to ionizing radiation when they undergo this examination. During the past decade, radiomics have developed rapidly not only in oncology studies but also for many other diseases due to its cost-effectiveness and non-invasive nature (Ren et al., 2021). Radiomics are the processes for high-throughput extraction of quantitative features that result in the conversion of images into mineable data and the subsequent analysis of these data for decision support. These are different from the traditional practices of treating medical images as pictures intended solely for visual interpretation and potentially improve diagnostic, prognostic, and predictive accuracy (Gillies et al., 2016). A recent study showed that radiomics features extracted from the image of coronary CT angiography act as a non-invasive tool for predicting chronic myocardial ischemia can help to identify high-risk patients with coronary artery disease (Shu et al., 2020). In our study, the patients with unilateral MCA occlusion were selectively collected. To our knowledge, whether the radiomics features based on routine CT images could reflect abnormal brain perfusion state has not been reported before.

Radiomics is a promising field in medical imaging research; however, the clinical implementation of radiomics has been challenging because of concerns about reproducibility. On general idea, the region of interest (ROI) drawing was based on the easily identifiable boundary of the anatomical structure or lesion area in previous radiomic research. Previous research (Leijenaar et al., 2013) reported the variability according to the enlargement of the lesion area within five different independent tumor delineations by multiple observers, the results showed that the majority of assessed features had both a high test-retest and interobserver stability in terms of their intraclass correlation coefficient (ICC), which means the interobserver differences in delineations affected feature reproducibility to some degree. Park et al. (2021) reported that MR radiomic features showed good robustness with different slice thicknesses and pixel spaces in patients with cervical cancer. These researches focused on the same task with different methods, but the outline of the ROIs was roughly similar in shape, and with good reproducibility, especially in the first-order feature. In actuality, the boundary of MCA territory on routine CT could not be easily recognized, even in previous CTP researches the drawing of MCA territory was still inconsistent (Kim et al., 2000; Trojanowska et al., 2006; Shi et al., 2021). In this study, we proposed two quite different ROI drawing methods to verify the stability of our results.

## Materials and Methods

### Data Collection

We obtained study approval from the ethics committee of the Qilu Hospital (Qingdao, Shandong Province, China) with a waiver for obtaining informed consent from the patients. We retrospectively analyzed data of patients who underwent both brain precontrast CT and CTP at our hospital from April 2015 to July 2021. Inclusion criteria were as follows: (1) severe stenosis or occlusion in unilateral MCA certified by digital subtraction angiography (DSA), CT, or MR angiography; (2) CTP showed the MCA blood supply area with deficit perfusion (prolonged mean transit time with or without decreased CBF); (3) without obvious abnormal finding or artifacts; and (4) angiography, CTP, and routine CT were finished in 2 weeks. Exclusion criteria were as follows: (1) severe stenosis or occlusion of contralateral MCA or internal carotid artery and (2) obvious low-density areas in the brain parenchyma. At last, a total of 52 cases of patients were included in this study and all the samples were randomly divided into the training (41 cases, 78.8%) and testing (11 cases, 21.2%) sets. The testing set was only independently used for the model evaluation and comparison.

### Scanning Parameters

All the patients were scanned on the Siemens Definition Flash 64-slice CT scanner (Siemens, Somatom, Germany). For routine CT, scanning parameters were as follows: spiral scanning type with scan field-of-view 30 cm; tube current–time products, 320 mAs; tube voltage, 120 kVp; and matrix size 512 × 512. For CTP, scanning parameters were as follows: dynamic volume scanning with 21 phases of 40 s scanning time; scan field-of-view 30 cm; tube current–time products, 140 mAs; tube voltage, 80 kVp; slice thickness, 1.0 mm; matrix size 512 × 512; and the CTP was scanned 5 s after intravenous bolus injection of non-ionic iodine contrast agent with 5 ml/s injection rate (0.6 ml/kg body weight, Ultravist 300, Bayer, Germany) with 40 ml normal saline after that.

### Region of Interest Segmentation and Radiomic Feature Extraction

**FIGURE 1:**
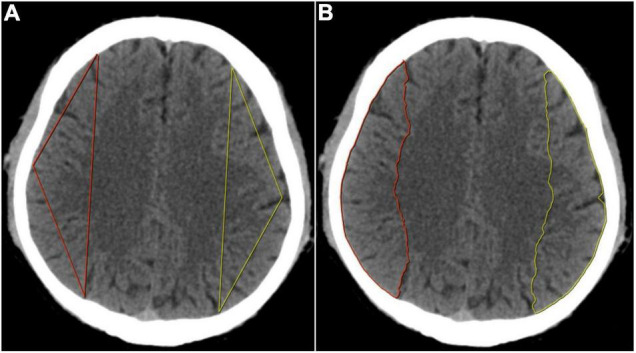
**(A)** Determination of the three vertexes in triangular contour was selected in the semioval center level. The anterior vertex was selected at the intersection of the longitudinal centerline of the unilateral cerebral hemisphere and the frontal cortex, the posterior vertex at the parietal cortex, and the middle vertex was selected at the midpoint of the convex surface of the brain, then the three points were connected in a straight line to form a triangular contour. **(B)** Determination of the semiautomatic contour was also selected in the semioval center level. The anterior and posterior points were selected with the same method as triangular contour and then the two points were automatically connected and the convex edge of the cerebral hemisphere was automatically outlined to form a semiautomatic contour.

We downloaded the original Digital Imaging and Communications in Medicine (DICOM) images of routine CT sequence and imported them to the Deepwise multimodal research platform (V1.6.2)^1^ for image annotation. One experienced radiologist (PAn) manually sketched a triangular contour and a semiautomatic contour of the MCA blood supply area at the semioval center slice, as shown in Figure 1. We chose the centrum semiovale as the standard slice for ROI drawing which is the classic level for measuring cerebral ischemic changes in the MCA territory according to the previous study (van Everdingen et al., 2000), and used a triangular-contour ROI drawing method trying to avoid the cortical areas sensitive to cerebral perfusion changes (Virley et al., 2004; Kazumata et al., 2017), to verify our hypothesis that imaging omics can also detect the perfusion differences in the white matter areas of both hemispheres that are not sensitive to perfusion changes. Intelligent Scissors (Mortensen and Barrett, 1998) algorithm was implemented as our semiautomatic segmentation method which can quickly locate the edge of the image area at runtime, and more accurately complete the outline and segmentation of the entire ROI by interacting with the user.

To utilize the information in the image as much as possible, data were preprocessed by 8 kinds of filters (wavelet, Laplacian of Gaussian, square, square root, logarithm, exponential, gradient transform, and local binary pattern transform) instead of just using the original image. Respectively, we extracted radiomic features from the two-dimensional slices of the triangular and the semiautomatic sketching ROI. For each delineation method, a total of 1,379 features were extracted, namely, 270 first-order features, 14 shape features, 330 gray level co-occurrence matrix (GLCM) features, 240 gray level size zone matrix (GLSZM) features, 240 gray level run length matrix (GLRLM) features, 210 gray level dependence matrix (GLDM) features, and 75 neighboring gray-tone difference matrix (NGTDM) features. For the training set, the values of each feature were standardized using Z-score and the means and the variances of them were reused for the standardized procedures on the testing set.

### Feature Selection and Model Construction

The ANOVA was performed to filter the extracted radiomic features (*p* ≤ 0.1). It is a classical statistical technique that is used to compare the differences among means based on *F*-test. And then, elastic net regression with fivefold cross-validation (CV) was conducted to further filter and choose the optimized subset of features in the training set. Elastic net overcomes the limitations of the least absolute shrinkage and selection operator (LASSO) by combining its *L*_1_ penalty with the *L*_2_ penalty of the ridge regression. After that, we implemented 9 different machine learning algorithms—logistic regression (LR), support vector machine (SVM), K-nearest neighbors (KNN), linear discriminant analysis (LDA), quadratic discriminant analysis (QDA), Gaussian naive Bayes (GNB), artificial neural network (ANN), random forest (RF), and XGBoost and CatBoost—in the training dataset and tuned the hyperparameters of these models using grid search method and fivefold CV. We used the receiver operating characteristic (ROC) analysis to evaluate the performance of the algorithms to determine the optimal one. And then the best models of triangular contour and semiautomatic contour were retrained in the whole training set to establish the radiomics score (rad-score) and evaluated in the testing set. In the training set, Youden index (Youden, 1950) analysis was constructed to determine the optimal threshold (cutoff value) of classification probability which was then used to predict in the testing set. Metrics like the area under the ROC curve (AUC), accuracy, F1 score, sensitivity, and specificity were calculated. Finally, we used the decision curve and calibration curve to measure the clinical usefulness of the model.

### Statistical Analysis

When the differences between the samples from the two groups were normally distributed, we performed the paired *t*-test; otherwise, the Wilcoxon signed-rank test was performed. In addition, since the scale of the testing set is quite small, the paired permutation test was applied. All the statistical analyses were performed using Python programming language (version 3.8.8)^2^ and its open-source package. First, the “PyRadiomics” package was used to extract radiomics features from the original images and transformed images. Second, the tools in “Scikit-learn” were used to implement the machine learning algorithm, perform the fivefold CV and construct the rad-score. Third, all visualizations were done using packages “Matplotlib” and “Seaborn.” At last, we use the classic statistical library “Stats” from “Scipy” to complete all the tests.

## Results

### Demographic Characteristics

The clinical and demographic characteristics of the subjects are shown in Table 1. Unilateral MCA occlusion can be seen not only in the elderly, but also in young and middle-aged people. Most of the patients have clinical symptoms, such as dizziness, fatigue, limb numbness, or bradylalia.

**TABLE 1:** The clinical and demographic characteristics of the subjects.

Clinical and demographic characteristics	Statistical value
Age (y, mean ± SD, range)	59.48 ± 13.01 (32–87)
Gender (male/female)	26/26
Symptoms (dizziness, fatigue, limb numbness, or barylalia, yes/no)	45/7

### Radiomics Signature Construction and Validation

**FIGURE 2:**
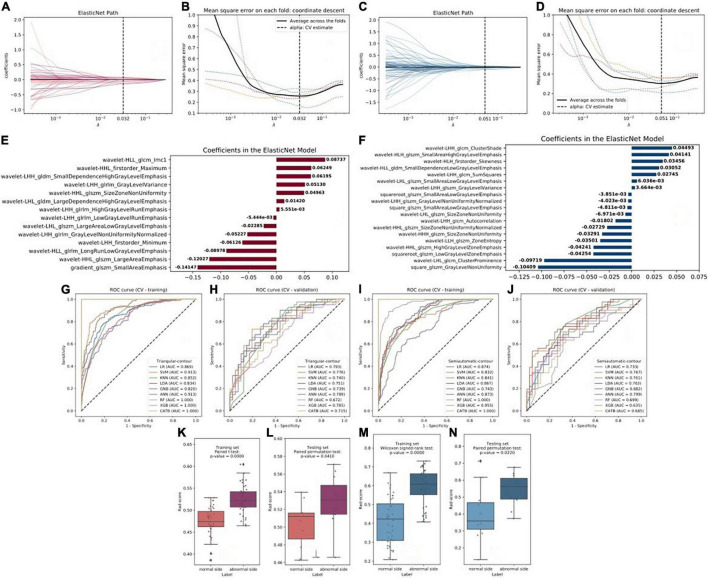
**(A,C)** Radiomics feature selection and rad-score construction of triangular contour (red, left) and semiautomatic contour (blue, right). The coefficient lambda of the penalty term in elastic net was seen as a hyperparameter and tuned *via* the fivefold cross-validation (CV) method. The black curve showed the average mean square error (MSE) for each model given lambda. The x-axis indicated the values of lambda. The vertical lines marked the values of the best lambda which were 0.032 and 0.051. **(B,D)** Radiomics features coefficients reduction-path curves. Finally, 14 non-zero factors for triangle contour and 19 for semiautomatic contour were selected. **(E,F)** The retained non-zero-coefficient features were plotted on the y-axis and their coefficients in the elastic net were plotted on the x-axis. **(G–J)** The receiver operating characteristic (ROC) curves of the 9 radiomics machine learning models. **(K–N)** Paired samples tests showed that there were significant differences in the rad-score which would be used to discriminate the patients into the two classes.

At first, a total of 1,379 radiomic features were extracted from each cerebral hemisphere of the subject. In the training set, the ANOVA was performed to select the features that were significantly different between vascular stenosis side class and normal side class and 57 features were retained for triangle contour and 86 features were retained for semiautomatic contour. After screening by the elastic net regression method with fivefold CV, 14 features were retained for triangle contour and 19 features were retained for semiautomatic contour (Figures 2A–D). The features with non-zero coefficients are shown in Figures 2E,F and the weight of each feature that contributed to the established signature was also displayed. Figures 2G–J and Table 2 illustrate the performance of 9 machine learning algorithms. In the triangle-contour part, LR, with an L_2_ penalty term whose coefficient was 28.031, had the best performance because its AUC (0.793) was highest in the CV phase and its AUC (0.869) in CV training indicated without overfitting. For semiautomatic contour, ANN, which had the learning rate of 0.000812, a penalty term whose coefficient is 19.490, and just one hidden layer with 100 units, was considered the optimal model for the same reason (0.873 AUC in the CV-training phase and 0.799 AUC in the CV). Based on these two best algorithms with optimal hyperparameters, the classifiers were retrained using the whole training data and radiomics signatures were established, respectively.

**TABLE 2:** Performance of 9 machine learning models in the fivefold CV training and validation phase.

Model	CV-training						CV-validation					
	AUC (95% CI)	SD	Accuracy	F1-score	Sensitivity	Specificity	AUC (95% CI)	SD	Accuracy	F1-score	Sensitivity	Specificity
**Triangular-contour**												
LR	0.869 (0.837–0.901)	0.019	0.796	0.774	0.817	0.791	**0.793 (0.714–0.871)**	0.050	0.744	0.780	0.707	0.753
SVM	0.913 (0.887–0.938)	0.016	0.857	0.787	0.927	0.846	0.776 (0.691–0.864)	0.054	**0.768**	0.732	0.805	0.759
KNN	0.852 (0.816–0.886)	0.021	0.787	0.793	0.780	0.788	0.740 (0.657–0.832)	0.053	0.707	**0.927**	0.488	0.760
LDA	0.834 (0.797–0.870)	0.022	0.750	0.738	0.762	0.747	0.751 (0.657–0.840)	0.056	0.695	0.805	0.585	0.725
GNB	0.820 (0.783–0.860)	0.023	0.738	0.762	0.713	0.744	0.739 (0.648–0.830)	0.055	0.707	0.561	0.854	0.657
ANN	0.913 (0.888–0.936)	0.015	0.835	0.829	0.841	0.834	0.789 (0.711–0.866)	0.048	0.732	0.902	0.561	**0.771**
RF	1.000 (1.000–1.000)	0.000	1.000	1.000	1.000	1.000	0.672 (0.576–0.773)	0.060	0.634	0.390	**0.878**	0.516
XGB	1.000 (1.000–1.000)	0.000	1.000	1.000	1.000	1.000	0.785 (0.695–0.864)	0.050	0.732	0.659	0.805	0.711
CATB	1.000 (1.000–1.000)	0.000	1.000	1.000	1.000	1.000	0.715 (0.620–0.807)	0.056	0.659	0.805	0.512	0.702
**Semiautomatic-contour**												
LR	0.874 (0.841–0.904)	0.019	0.787	0.817	0.756	0.793	0.733 (0.644–0.826)	0.055	0.683	0.561	0.805	0.639
SVM	0.832 (0.793–0.867)	0.022	0.759	0.689	0.829	0.741	0.767 (0.681–0.851)	0.051	0.720	0.537	0.902	0.657
KNN	0.841 (0.807–0.872)	0.020	0.759	0.774	0.744	0.763	0.761 (0.678–0.853)	0.054	0.720	**0.780**	0.659	**0.736**
LDA	0.867 (0.833–0.897)	0.020	0.780	0.738	0.823	0.771	0.763 (0.677–0.848)	0.052	0.720	0.732	0.707	0.723
GNB	0.743 (0.698–0.784)	0.026	0.695	0.640	0.750	0.677	0.682 (0.584–0.778)	0.060	0.683	0.659	0.707	0.675
ANN	0.873 (0.841–0.902)	0.019	0.799	0.732	0.866	0.784	**0.799 (0.718**–**0.876)**	0.048	**0.744**	0.537	**0.951**	0.677
RF	1.000 (1.000–1.000)	0.000	1.000	1.000	1.000	1.000	0.699 (0.604–0.793)	0.057	0.671	0.634	0.707	0.658
XGB	0.955 (0.937–0.972)	0.011	0.899	0.890	0.909	0.898	0.635 (0.532–0.737)	0.062	0.671	0.707	0.634	0.682
CATB	1.000 (1.000–1.000)	0.000	1.000	1.000	1.000	1.000	0.685 (0.585–0.787)	0.060	0.683	0.707	0.659	0.690

*SD, standard deviation of AUC; LR, Logistic Regression; SVM, Support Vector Machine; KNN, K-Nearest Neighbors; LDA, Linear Discriminant Analysis; GNB, Gaussian Naive Bayes; ANN, Artificial Neural Network; RF, Random Forest; XGB, XGBoost; CATB, CatBoost. Bold values highlight the best results of each indicator in different models in the validation set.*

### Comparison of Radiomic Score With Two Sketched Contour Methods

**FIGURE 3:**
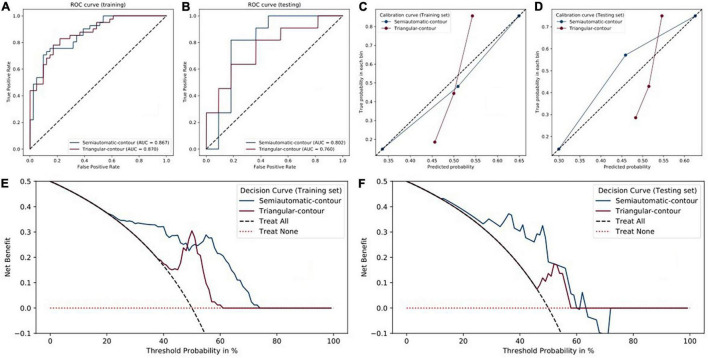
Performance of triangular-contour (red) and semiautomatic-contour (blue) models. **(A,B)** The ROC curves of the training and testing sets. **(C,D)** Calibration curves. **(E,F)** Decision curves.

For semiautomatic contour, the radiomics signature showed good predictive performance with an AUC value of 0.867 in the training set (Figure 3A) and a value of 0.802 in the testing set (Figure 3B). According to the optimal cutoff value of 0.568, the cerebral hemispheres were classified into vascular stenosis side class and normal side class. Both in the training and testing sets, rad-score was significant difference between in vascular stenosis side class and normal side class (Figures 2M,N).

For triangle contour, the radiomics signature showed good predictive performance with an AUC value of 0.870 in the training set and a value of 0.760 in the testing set. According to the best cut-off value of 0.507, the cerebral hemispheres were classified into vascular stenosis side class and normal side class. Figures 2K,L indicated that the rad-score was significant difference between in both the training set (*p* < 0.001) and validation set (*p* = 0.041).

Except for the sensitivity, in the testing set, the metrics of the semiautomatic-contour model were better than the triangular-contour model although the two classifiers had similar performances in the training set (Table 3). The ROC curves were analyzed by the DeLong test, which showed that there was no significant difference in both the training set and testing set (*P*-values are 0.3242 and 0.8057, respectively). As shown in Figures 3E,F, we used the decision curves to evaluate the clinical usefulness of the model. It showed that the semiautomatic-contour model would be more beneficial than the triangular counterpart. From the perspective of the calibration curve analysis (Figures 3C,D), the semiautomatic-contour model was closer to the ideal in both of the training and testing sets.

**TABLE 3:** Comparisons of radiomics models for semiautomatic and triangular contour in the training and testing set.

Model	AUC (95% CI)	*SD*	Accuracy	F1-score	Sensitivity	Specificity	Threshold
**Training set**							
Semiautomatic-contour	0.867 (0.801–0.927)	0.039	0.805	0.784	0.707	0.902	0.568
Triangular-contour	0.870 (0.806–0.929)	0.038	0.805	0.800	0.780	0.829	0.507
**Testing set**							
Semiautomatic-contour	0.802 (0.616–0.971)	0.103	0.818	0.818	0.818	0.818	/
Triangular-contour	0.760 (0.580–0.923)	0.109	0.545	0.667	0.909	0.182	/

## Discussion

In this study, we investigated the difference of the two sides of the hemisphere based on routine CT in the patients with lateral MCA stenosis and the CTP showed abnormal cerebral perfusion in the vascular stenosis side. For semiautomatic contour, a radiomics signature constructed by 16 predictive features in this study showed good predictive efficacy in distinguishing vascular stenosis side from normal side in the training set (AUC = 0.867, 95% CI: 0.801–0.927) and validated the testing set (AUC = 0.802, 95% CI: 0.616–0.971); for triangular contour, good predictive efficacy could also be found in the training set (AUC = 0.870, 95% CI: 0.806–0.929) and validated the testing set (AUC = 0.760, 95% CI: 0.580–0.923). Based on the Youden index for semiautomatic contour, the sensitivity and specificity of the radiomics signature in the testing set were both 81.8% for stenosis side vs. normal side, but for triangular contour, the specificity was low in the testing set.

Considerable interest has grown in the recent years concerning the role of chronic cerebral hypoperfusion on the development and progression of neurocognitive disorders (Ciacciarelli et al., 2020). But, it is not very clear whether the changes in CBF precede the neurovascular dysfunction, whether the hypoperfusion is a cause or a consequence, or just an epiphenomenon. Previous study has shown the susceptibility of different animal models to developing Alzheimer-like pathology under conditions of reduced cerebral perfusion (Farkas et al., 2007). For another aspect, epidemiological data showing the coexistence of vascular risk factors and cognitive disorders (Breteler et al., 1994), supported the causal relationship between cerebral vascular and neurodegenerative disorders. In large population-based studies, the CBF reduction precedes cognitive decline and hippocampal atrophy (Ruitenberg et al., 2005) as to suggest causality. Besides that, a significant amount of attention has been paid to using neuroimaging to assess potential benefits by identifying areas of ischemia that have not yet experienced cellular death. On general idea, the perfusion–diffusion mismatch is used as a simple metric for potential benefit with timely intervention, yet penumbral patterns provide an inaccurate predictor of clinical outcome (Feng et al., 2018). So as an important aspect, hypoperfusion is a metabolic state that should attract clinical attention. But CTP has its limitation for one of the important reasons is the radiation dose. Our results showed the radiomic feather based on routine non-contrast CT could distinguish stenosis side and normal side properly which has perfusion difference manifested by CTP.

Although radiomics had promising applications in many diseases and not only in tumor issues, a major obstacle to its clinical application is that the robustness of extracted radiomic features is not very clear. The research on the stability of radiomic features in CTP maps showed that the voxel size, image discretization, HU threshold, and temporal resolution have to be standardized to build a reliable predictive model based on CTP radiomics analysis (Bogowicz et al., 2016). On the other hand, interrater variability and stability of the extracted radiomic features are one of the limitations (Elshafeey et al., 2019), and it is difficult to standardize the parameters during image acquisition for all patients in clinical settings (Park et al., 2021). Such as, several studies have reported that pixel size resampling and interpolation improved reproducibility in CT radiomic features (Mackin et al., 2015; Shafiq-Ul-Hassan et al., 2017). In fact, the manual sketch is usually considered a poorly reproducible, boring, and time-consuming method in medical imaging. This situation imposed the development of autosegmentation methods. At present, most published methods have been optimized and validated on a specific, usually homemade dataset. The various methods under comparison often provide different segmentation. Therefore, the approaches combining different segmentation paradigms, either through consensus or by learning automatically to choose the most appropriate method appear as promising developments for the future (Hatt et al., 2018). In this study, we used two different shapes of ROI for radiomic feature extraction, our results showed both methods could distinguish the stenosis side and normal side which has perfusion difference manifested by CTP, but for triangular contour, the specificity was low in the testing set. We concluded that the autosegmentation method had more benefits than that of the manual method.

Based on MR perfusion images, radiomic features had shown good performance in differentiating pseudoprogression and progressive disease; on another aspect, the radiomic features of arterial spin labeling (ASL) and dynamic susceptibility contrast imaging-derived parameters (CBF) had a similar ability for low-grade gliomas (LGGs) and high-grade gliomas (HGGs) differential diagnoses (Hashido et al., 2020). MR perfusion was not only used in the tumor, previous animal tests showed the CBF of the neocortex decreased obviously than brain stem and hippocampus in hypothermic selective cerebral perfusion (Strauch et al., 2004). C-arm flat detector CT (FDCT) parenchymal blood volume (PBV) imaging showed the cerebral cortex had higher blood volume than cerebral white matter, the PBV values were relatively high for the white matter and relatively low for the cortical gray matter compared with PET results (Kamran and Byrne, 2016). Significant reductions in CBF were seen in all the white matter regions of radiological leukoaraiosis and clinical lacunar stroke patients, there was no significant difference in white matter CBV between cases and controls, but gray matter CBV was significantly higher in patients than in controls (Markus et al., 2000). In our study, based on different shapes of ROI for radiomic feature extraction, our results showed the AUC of autosegmentation methods was high than triangular contour in the testing set for distinguishing the stenosis side and normal side. Because the more cerebral gray matter was included in autosegmentation methods, we concluded that the effect of cerebral gray matter is greater in the state of hypoperfusion.

Several limitations in our primary and exploratory study should be noted: (a) the sample size is relatively small because of the low prevalence of MSA and the fact that this was a single-center study and (b) the retrospective study did not relate radiomic features to the patient’s clinical scales, such as the motor and activities of daily living (ADL) scale. Larger and more randomized samples are needed in the future.

Despite these limitations, we constructed a fine model using radiomic features to reflect the difference of the two sides of the hemisphere in the patients with lateral MCA stenosis. What is encouraging is that our results indicated that radiomics on precontrast CT images could reflect perfusion difference between left and right cerebral hemispheres to some extent.

## Data Availability Statement

The original contributions presented in the study are included in the article/supplementary material, further inquiries can be directed to the corresponding author/s.

## Ethics Statement

The studies involving human participants were reviewed and approved by the Ethics Committee of Qilu Hospital (Qingdao, Shandong Province, China). Written informed consent for participation was not required for this study in accordance with the national legislation and the institutional requirements.

## Author Contributions

QR drafted and submitted the manuscript. PA drew the ROIs and designed the idea. KJ and JX made the statistical analysis and the figure drawing. XX, ZH, CH, and QJ modified the manuscript. XM designed and modified the manuscript. All authors contributed to the article and approved the submitted version.

## Conflict of Interest

KJ, JX, and CH are employed by Deepwise AI Lab, Beijing Deepwise and League of PHD Technology Co., Ltd. The remaining authors declare that the research was conducted in the absence of any commercial or financial relationships that could be construed as a potential conflict of interest.

## Publisher’s Note

All claims expressed in this article are solely those of the authors and do not necessarily represent those of their affiliated organizations, or those of the publisher, the editors and the reviewers. Any product that may be evaluated in this article, or claim that may be made by its manufacturer, is not guaranteed or endorsed by the publisher.

## Footnotes

1
http://keyan.deepwise.com

2
http://www.python.org
